# The varying estimation of infertility in Ethiopia: the need for a comprehensive definition

**DOI:** 10.1186/s12905-024-03118-8

**Published:** 2024-05-08

**Authors:** Bilen Mekonnen Araya, Heather M. Aldersey, Saionara Camara, Kassahun Alemu, Silke Dyer, Maria P. Velez

**Affiliations:** 1 Department of Rehabilitation Science, School of Rehabilitation Therapy, Queen’s University, Kingston, Canada; 2 Department of Clinical Midwifery, School of Midwifery, University of Gondar, Gondar, Ethiopia; 3 School of Rehabilitation Therapy, Queen’s University, Kingston, Canada; 4 Department of Physical Therapy, Federal University of Rio Grande do Norte, Natal, Brazil; 5 Institute of Public Health, College of Medicine and Health Science, University of Gondar, Gondar, Ethiopia; 6 Department of Obstetrics & Gynaecology, Faculty of Health Sciences, Groote Schuur Hospital, University of Cape Town, Cape Town, South Africa; 7 Department of Obstetrics and Gynaecology, School of Medicine, Queen’s University, Kingston, Canada

**Keywords:** Infertility, Definition, Estimation, Ethiopia, Africa, DHS, Demographic, Current duration

## Abstract

**Background:**

Infertility is a marginalized sexual and reproductive health issue in low-resource settings. Globally, millions are affected by infertility, but the lack of a universal definition makes it difficult to estimate the prevalence of infertility at the population level. Estimating the prevalence of infertility may inform targeted and accessible intervention, especially for a resource-limited country like Ethiopia. This study aims to estimate the prevalence of female infertility in Ethiopia using the Demographic and Health Survey (DHS) through two approaches: (i) the demographic approach and (ii) the current duration approach.

**Methods:**

Data from 15,683 women were obtained through the 2016 Ethiopian DHS. The demographic approach estimates infertility among women who had been married/in a union for at least five years, had never used contraceptives, and had a fertility desire. The current duration approach includes women at risk of pregnancy at the time of the survey and determines their current length of time-at-risk of pregnancy at 12, 24, and 36 months. Logistic regression analysis estimated the prevalence of infertility and factors associated using the demographic approach. Parametric survival analysis estimated the prevalence of infertility using the current duration approach. All estimates used sampling weights to account for the DHS sampling design. STATA 14 and R were used to perform the statistical analysis.

**Results:**

Using the demographic definition, the prevalence of infertility was 7.6% (95% CI 6.6–8.8). When stratified as primary and secondary infertility, the prevalence was 1.4% (95% CI 1.0-1.9) and 8.7% (95% CI 7.5–10.1), respectively. Using the current duration approach definition, the prevalence of overall infertility was 24.1% (95% CI 18.8–34.0) at 12-months, 13.4% (95% CI 10.1–18.6) at 24-months, and 8.8% (95% CI 6.5–12.3) at 36-months.

**Conclusion:**

The demographic definition of infertility resulted in a lower estimate of infertility. The current duration approach definition could be more appropriate for the early detection and management of infertility in Ethiopia. The findings also highlight the need for a comprehensive definition of and emphasis on infertility. Future population-based surveys should incorporate direct questions related to infertility to facilitate epidemiological surveillance.

**Supplementary Information:**

The online version contains supplementary material available at 10.1186/s12905-024-03118-8.

## Background

Infertility is a disease that generates disability as an impairment of function [1]. Clinically, infertility is defined as a reproductive system disease that results in the failure to achieve a clinical pregnancy after 12 months or more of regular unprotected sexual intercourse [2, 3]. Infertility can be classified as primary or secondary. Primary infertility occurs when a woman is unable to ever bear a child, either due to the inability to become pregnant or carry a pregnancy to live birth. Secondary infertility is the failure to become pregnant or carry a pregnancy to live birth after a previous pregnancy or the ability to carry a pregnancy to live birth [4]. Infertility is considered a public health issue [5] as it affects over 10% of women globally and is associated with preventable causes (i.e., advanced maternal age, lifestyle factors, and sexually transmitted infections) [6–9].

Infertility is a marginalized sexual and reproductive health issue [7, 10], with most infertile couples residing in developing countries [11]. An estimated 48 million couples and 186 million individuals live with infertility globally, while 34 million women are predominantly from developing countries [12–15]. Africa has an “infertility belt” that stretches across central Africa. This concept suggests that in this region, infertility is often most prevalent where fertility rates are also high [16–19]. The prevalence of infertility is difficult to determine. Different units of analysis – women, men, couples, or both – are also used interchangeably or without precision [20–22]. Population-based surveys show that primary infertility ranges from 0.6 to 49.9%, while secondary infertility is estimated at 4.8 to 49.8% [12, 20, 23–26]. A recent study by the World Health Organization (WHO) highlights the considerable variation in estimates of infertility, partly explained by differences in how infertility is defined and measured among studies and the high prevalence of infertility [27]. The high variation range in the estimation observed may be due to the difference in the measured outcome, units of analysis, period of exposure to pregnancy, and study design, among other factors [7, 12–14, 21, 25]. Determining infertility estimates are necessary to highlight the true burden of infertility and, thus, access to care [12, 28]. Estimating infertility helps deliver targeted interventions by the health care system and reproductive policymakers [13, 21], especially for a resource-limited country such as Ethiopia.

From the different approaches used to estimate fecundability (i.e., the probability of conceiving in any given menstrual cycle), prospective cohort studies that collect information about time to pregnancy (TTP) are considered the gold standard. However, this study design is expensive and not feasible at a national population level [7, 29]. Approaches that include all individuals at risk of pregnancy through national surveys – such as the demographic and current duration approaches – are preferable, especially in low-resource settings. The demographic and current duration methods use household surveys that do not directly collect data on the time a couple is trying to conceive. However, these surveys gather other related data that can infer TTP [13, 20]. The use of these two approaches may help to understand differences with other studies and improve the overall characterization of infertility at the population-level [30]. They allow cost-effective population-based estimates and cover all couples who are at risk of pregnancy. They can be easily implemented using robust population-based databases from Low and Middle-Income Countries (LMIC) like the Demographic and Health Surveys (DHS). Furthermore, these approaches are commonly used in other population-based studies worlwide, which makes it easier to compare results for epidemiological surveillance [7, 27]. In this study, there will be no comparison of estimates, beyond general comments, due to the different timelines, eligibility criteria, methods of analysis, and assumptions used by the two approaches [27].

Limited studies in Ethiopia estimate the magnitude of infertility [31–33]. The limited studies are outdated, gray, and cover a small area. Our study uses the latest available national data to estimate infertility. Thus, this study primarily aimed to examine the differences in the estimate of infertility among women in Ethiopia using two different approaches: the demographic and current duration approach. The demographic approach estimates infertility prevalence among women who have been married or in a union for at least five years and not had a live birth, ever for primary infertility or ≥ 5 years ago for secondary infertility. In contrast, the current duration approach determines the length of time-at-risk of pregnancy for women at risk of pregnancy at 12, 24, and 36 months. As a secondary objective, we examined factors associated with infertility.

## Methods

### Study population

Both the demographic and current duration approaches were estimated using the 2016 Ethiopian Demographic Health Survey (EDHS). The 2016 EDHS is the fourth population-based survey implemented by the Central Statistical Agency (CSA) [34]. This study used data from questionnaires developed by the DHS program, which can be found here (https://dhsprogram.com/Methodology/Survey-Types/DHS.cfm). Demographic and Health Surveys (DHS) are nationally representative household surveys that provide data for a wide range of monitoring and impact evaluation indicators in the areas of population, health, and nutrition. More specifically, the questionnaire for the EDHS is found at the end of the EDHS 2016 report [34]. Five questionnaires were used for the 2016 EDHS: the household questionnaire, the woman’s questionnaire, the man’s questionnaire, the biomarker questionnaire, and the health facility questionnaire. The survey targeted women aged 15–49 and men aged 15–59 in randomly selected households across Ethiopia and collected detailed information on background characteristics, sexual and reproductive health issues, and other related issues for the past five years before the survey was conducted. The women’s questionnaire was used for this study where a total of 15,683 women participated.

#### The demographic approach

##### Infertility definition

Overall infertility was defined as the combination of primary and secondary infertility. Primary infertility was defined as the proportion of women aged 20–49 who were married or in a union at the time of data collection or had been for the past five years, had not ever used any form of contraceptive, who had a fertility desire and had not ever delivered a live birth. (See Additional file 1)

Secondary infertility was defined as the proportion of women aged 20–49 who were married or in a union at the time of data collection or had been for the past five years, who had a history of giving live birth (before five years of the survey), who had a fertility desire for an additional child(ren) during that time, had not used any form of contraceptive methods after giving birth before five years and had not given birth within five years of the survey. (See Additional file 2)

When applying these definitions, the following aspects deserve attention for the demographic approach.

**Table:** 

Age	20–49 years	Women under 20 were excluded to minimize adolescent infecundity.
Women’s union status	Married, or in-union	Married or in a union can be a proxy for regular sexual intercourse. The union time on EDHS is calculated starting from the first union, making it difficult to know the time for women with multiple partnerships. Thus, we first selected women whose first union was five or more years prior to the interview, and from that subset, we drew out those who were married or in a union at the time of data collection.
Exposure time	5- years	Five years consider the time from pregnancy to birth which minimizes the bias of misclassifying pregnant women as infertile.
Contraceptive use	Yes	Not using a contraceptive serves as a proxy for unprotected sexual intercourse. The EDHS data only states the start of contraceptive use, not the discontinuation time. Thus, ever contraceptive use history was used to capture current use as well for primary infertility estimates. For secondary infertility estimate, we used the contraceptive calendar and included contraceptive use for the past five years after the live birth. Those using outside the calendar were also considered non-users to ensure usage reliability. The calendar is a month-by-month history of women’s reproduction and contraceptive use for a period of 5 years before the survey.
	No	
Fertility desire	Yes	A desire for a child(ren) serves as a proxy for unprotected sexual intercourse. Also, it helps to exclude voluntary infertility.
	No	
Live birth	Yes	Live birth is the desired individual and social outcome among couples rather than pregnancy. Also, miscarriage, abortion, and stillbirth are prone to recall bias, misclassification and are sensitive for women to respond to population-based surveys.
	No	

#### Analysis

Bivariate and multivariable logistic regression with a 95% confidence interval (CI) was fitted to estimate infertility. The factors associated were identified from other studies [5, 32, 35, 36] and are grouped as: (i) women’s sociodemographic variables, (ii) women’s health-related variables, and (iii) partners’ characteristics. The final model included variables with a *p*-value < 0.20 from the bivariate analysis. Backward stepwise regression was used to determine significant variables with a *p*-value ≤ 0.05. All estimates used sampling weights to account for the survey design and were conducted using STATA 14.

#### The current duration approach

The current duration approach focuses on the time to pregnancy (TTP) from when a couple starts to attempt pregnancy until the pregnancy occurs. Assessing TTP at the population level requires asking women retrospectively about the time it took to become pregnant [7]. To date, the Demographic Health Survey (DHS) does not include information about TTP for couples trying to conceive or those with prior pregnancies. However, Polis and colleagues [7] proposed a statistical model to estimate TTP using DHS and hence were able to apply the current duration approach to estimate infertility using DHS data. We replicated Polis and colleagues’ [7] method, and the following is a summary of the assumptions they used. The population sampled based on eligibility for the current duration approach includes women ‘at risk’ of pregnancy at the time of the interview, defined as women who were 18–44 years old, married or cohabitating, sexually active within the past four weeks, and not currently using contraception (and had not been sterilized). The exclusion included women who: were currently pregnant at the time of data collection, had given birth in the past three months or were postpartum amenorrheic; had used depot medroxyprogesterone acetate within the last ten months; were menopausal or had a hysterectomy; had never menstruated, or were missing information on the timing of first sexual intercourse with a current partner.

##### Current Duration (CD) measure

For each individual, we calculated time-at-risk of pregnancy (a CD value), based on the following self-reported information: date of last live birth or pregnancy, duration of postpartum abstinence and amenorrhea for the most recent live birth, last contraceptive use, first cohabitation or intercourse with current partner, and date of interview. We excluded respondents with CD values < 0 (i.e. time not at risk of pregnancy). To account for the potential under-reporting of first-trimester pregnancies, data from the interview month and the preceding two months were excluded, as recommended by Polis and colleagues (https://osf.io/5jksy) (Additional file 3). This generates CD values spanning from the start of exposure to pregnancy risk up to 3 months prior to interview (i.e. a 3-month lag on CD values).

Parametric survival analysis, weighted to account for survey design, and assuming a generalized gamma distribution, were employed to estimate infertility at 12, 24, and 36 months with 95% CI calculated using 500 bootstrap samples. The analyses were censored after 36 months. In addition, multiple subgroup analyses were done to understand the infertility rates in different groups. The analytic sample and current duration measures were derived using STATA 14, and models were fitted using R software.

## Results

### The demographic approach

The overall infertility rate was 7.6% (95% CI: 6.6–8.8). The primary and secondary infertility rates were 1.4% (95% CI: 1-1.9) and 8.7% (7.5–10.1), respectively. Table 1 shows the distribution of sociodemographic characteristics for overall and by primary and secondary infertility. The mean age at the time of the survey (2016) was higher for female and male partners in infertile unions than those without infertility. When stratified by primary or secondary infertility, the mean age was similar in the primary infertility group but different in those with secondary infertility. The partner’s mean age was higher in the infertile unions than in the fertile unions across all categories. Education was comparable in the overall and secondary infertility samples. However, more educated women were in the primary infertility category than the fertile union. Across the three categories, there is a similarity in the women’s working status: more working women are infertile compared to fertile unions. On the other hand, across the three categories, more infertile women were found under those partners who were not working.

**Table 1:** Percent distribution (weighted) of independent variables by type of infertility based on the demographic approach

	Overall infertility status (8,253)			Primary infertility (8,253)			Secondary infertility (5,920)		
	Fertile union (*n* = 7,625)	Infertile union (*n* = 628)	*P*	Fertile union (*n* = 8,139)	Infertile union (*n* = 114)	*P*	Fertile union (*n* = 5,406)	Infertile union (*n* = 514)	*P*
**Age**, *mean*	32.8 (32.5; 33.1)	38.3 (37.4; 39.1)	< 0.001	33.2 (32.9; 33.5)	32.4 (30.5; 34.3)	0.44	31.9 (31.6; 32.2)	39.6 (38.8; 40.4)	< 0.001
**Age group**, *%*			< 0.001			0.53			< 0.001
20–29	35.9	13.5		34.1	41.9		38.2	7.2	
30–39	43.6	38.6		43.4	40.3		48.6	38.2	
40–49	20.4	47.9		22.5	17.8		13.2	54.6	
**Partner’s age**, *mean*	40.8 (40.4; 41.2)	48.4 (47.1; 49.8)	< 0.001	41.4 (40.9; 41.8)	43.9 (40.6; 47.2)	0.14	39.8 (39.4; 40.3)	49.4 (48; 50.9)	< 0.001
**Education**, *%*			0.98			0.04			0.81
No education	69.8	69.9		70	55.8		73.7	73	
Primary or higher	30.2	30.1		30	44.2		26.3	27	
**Partner’s education**^**a**^, *%*			0.17			0.76			0.26
No education	50.5	55.4		50.9	48.4		52.9	57	
Primary or higher	49.5	44.6		49.1	51.6		47.1	43	
**Currently working**, *%*			0.27			0.14			0.13
No	69	65.9		68.9	58.6		71.9	67.5	
Yes	31	34.1		31.1	41.4		28.1	32.5	
**Partner currently working**^**b**^, *%*			< 0.001			0.37			< 0.001
No	7.6	15.8		8.1	12		7.8	16.7	
Yes	92.4	84.2		91.9	88		92.2	83.3	
**Wealth index**, *%*			0.02			0.01			< 0.001
Poorest/poorer	40.42	34.25		40.09	30.36		44.86	35.1	
Middle/richer	41.2	39.9		41.2	32		40.8	41.6	
Richest	18.4	25.9		18.7	37.6		14.3	23.3	
**Type of residence**, *%*						0.01			< 0.001
Urban	13.7	20.5	0.01	14	30.8		10	18.2	
Rural	86.3	79.5		86	69.2		90	81.8	
**Number of unions**, *%*			< 0.001			0.02			< 0.001
1	80.6	70.1		80	66.2		82.1	70.9	
2 or more	19.4	29.9		20	33.8		17.9	29.1	
**Number of children**, *%*			< 0.001			< 0.001			0.02
0 to 3	36.3	50.3		36.5	100		31.3	39.2	
4 or more	63.7	49.7		63.6	0		68.7	60.8	
**Lifetime number of sex partners**, *%*			< 0.001			< 0.001			< 0.001
1	78.2	63.2		77.4	53.5		80	65.3	
2 or more	21.8	36.8		22.6	46.5		20	34.7	
**Age at first cohabitation**, *%*			< 0.001			< 0.001			0.13
< 20 years	83.4	75.1		83.1	57.9		83.4	78.9	
≥ 20 years	16.6	24.9		16.9	42.1		16.7	21.1	
**BMI**, *%*			< 0.001			0.19			< 0.001
Underweight (< 18.5)	18.3	23.3		18.8	12.3		19	25.7	
Normal (18.5–24.9)	73.8	65.8		73.2	72.8		74.6	64.3	
Overweight/ obese (> 25)	7.9	10.9		8	14.9		6.4	10	

a: primary and overall infertility (65 missing values), secondary infertility (44 missing values). b: Primary and overall infertility (90 missing values), secondary infertility (59 missing values)

Table 2 demonstrates the factors that were associated with overall, primary, and secondary infertility in the final model. Women aged 40–49 years had higher odds of overall infertility than those aged 20–29 years. When categorized by primary and secondary infertility, women aged 40–49 had lower odds of primary infertility than those aged 20–29, while older women had higher odds of secondary infertility than younger ones. Each year increase on partner’s age, was associated with increased odds of overall, primary, and secondary infertility. Women whose partners worked had lower secondary infertility odds than women whose partners were not working. While the wealth index was not associated with overall infertility, those in the richest index had higher odds of primary and secondary infertility than those in the poorest/poorer. Women with two or more unions in their lifetime had twice the odds of primary infertility compared to women with only one union. Age of first cohabitation was associated with higher odds of primary infertility, but lower odds of secondary infertility. Underweight (BMI < 18), was associated with overall and secondary infertility, but not with primary infertility. Smoking was associated with secondary infertility.

**Table 2:** Logistic regression for factors associated with infertility based on the demographic approach

Variables	Categories	Overall Infertility OR (95% CI)	Primary Infertility OR (95% CI)	Secondary Infertility OR (95% CI)
Women’s age	20–29	Ref	Ref	Ref
	30–39	3.37 (2.05; 5.51)	0.34 (0.14; 0.84)	7.42 (4.55; 12.12)
	40–49	9.57 (5.46; 16.78)	0.17 (0.05; 0.54)	48.06 (26.91; 85.74)
Partner’s age, mean (SD)	NA	1.03 (1.02; 1.05)	1.04 (1.01; 1.08)	1.04 (1.02; 1.05)
Partner currently working	No	Ref	-	Ref
	Yes	0.52 (0.37; 0.73)	-	0.48 (0.33; 0.70)
Wealth index	Poorest/poorer	Ref	Ref	Ref
	Middle/richer	-	1.09 (0.59; 2.01)	1.17 (0.83; 1.65)
	Richest	-	2.66 (1.27; 5.60)	1.92 (1.19; 3.08)
Number of unions	1	-	Ref	-
	2 or more	-	2.20 (1.15; 4.19)	-
Number of children	0 to 3	Ref	-	Ref
	4 or more	0.18 (0.13; 0.24)	-	0.13 (0.09; 1.19)
Age at first cohabitation	< 20 years	-	Ref	Ref
	≥ 20 years	-	4.03 (2.25; 7.22)	0.56 (0.37; 0.85)
BMI	Underweight (< 18.5)	1.37 (1.02; 1.82)	-	1.57 (1.13; 2.18)
	Normal (18.5–24.9)	Ref	-	Ref
	Overweight/obese (> 24.9)	1.02 (0.62; 1.68)	-	0.97 (0.57; 1.65)
Smoking	No	-	-	Ref
	Yes	-	-	4.06 (1.13; 14.61)

### The current duration approach

After applying the exclusion criteria for the CD approach, 14,106 women were not eligible for the CD while 1,577 were included in the analysis (Additional file 4). Relative to non-eligible women, those eligible were older, more were married, had prior children, reside in rural areas, had no education, and never used contraception. Of the eligible group for the current duration, 40.1% were in the 25–34 age group, 99% were married, and 12.9% were nulliparous. Rural dwellers comprised 86.5% of the sample, and the majority (66.9%) had no education. 15% had a history of pregnancy termination. While almost all women knew modern contraception methods (98%), only 28.8% had ever used contraception methods. Polygynous relationship – having more than one wife – was reported by 14.1% of the current duration sample, and the majority had more than 95 times sexual frequency in the last year of the survey with the most recent partner (67.1%). While 39.3% of women expressed that they wanted to have children within two years, 30.4% were not interested in a future pregnancy. Approximately 30% of the husbands/partners wanted more children than their wives.

Additional file 5 compares the demographics based on parity and fertility intentions for the CD sample. Parous women were older, the majority had no education, were in a polygynous relationship, had frequent sexual intercourse, and had less fertility desire within the next two years. Additional analysis demonstrated that most of those who wanted another pregnancy in the short term were aged 25–34 years (42.2%), the majority had no education (67.4%), were in a monogamous relationship (90.2%), and had frequent sexual intercourse (64.7%). In addition, 77.1% did not have a correct knowledge of the fertile period.

**Fig. 1:**
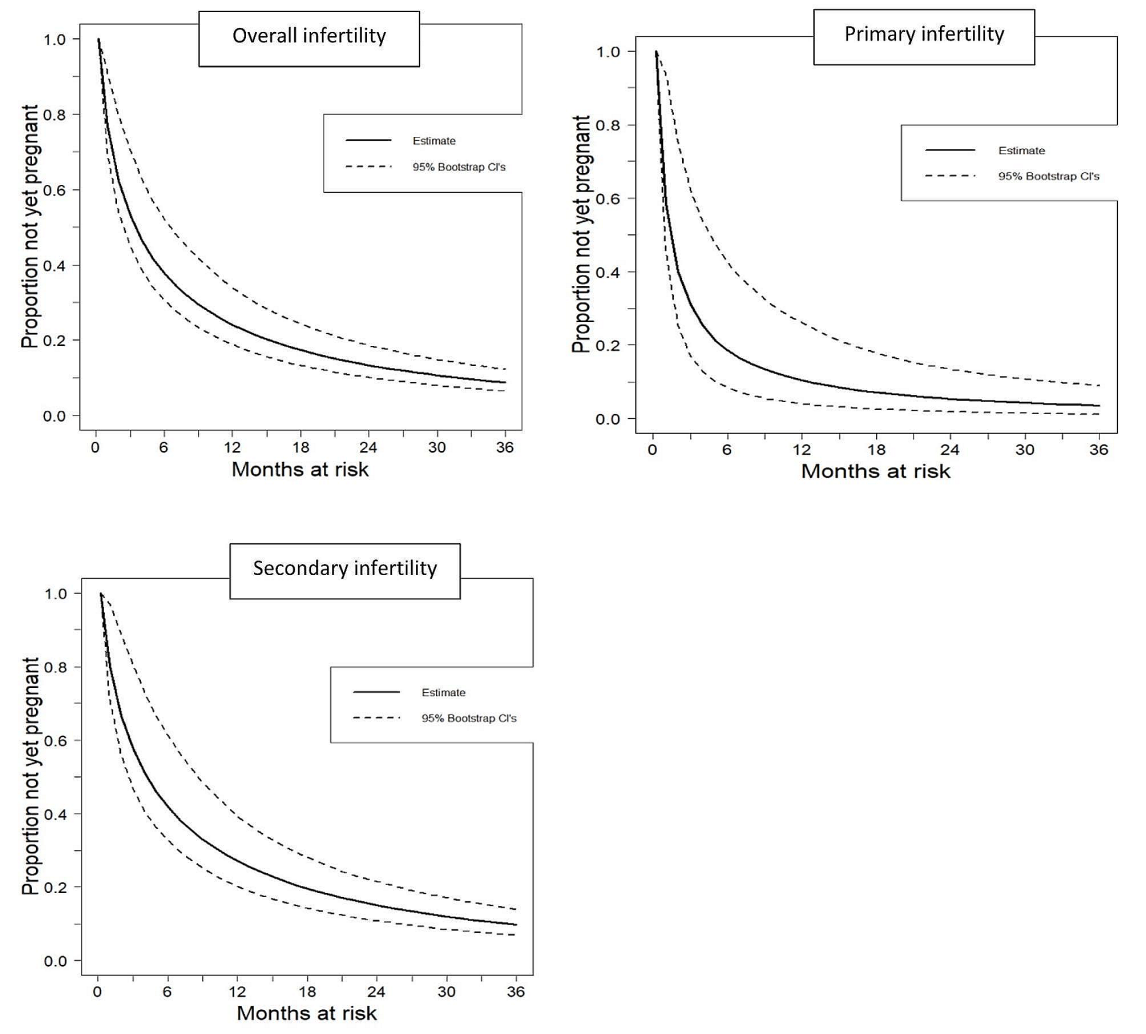
Survival function for time to pregnancy or end of pregnancy attempt estimated using a current duration approach

The estimates of infertility for different sub-groups for 12, 24, and 36 months are illustrated in Table 3; Fig. 1. The overall infertility estimate was 24.1% (95% CI: 18.8–34.0) at 12 months. For primary infertility, the 12-month infertility estimate was 10.4% (95% CI: 4.1–26.1), while it was higher for secondary infertility, 27.1% (95% CI: 20.2–39.4). The 24 months overall infertility estimate was 13.4% (95% CI: 10.1–18.6), with primary infertility at 5.4% (95% CI: 1.9–13.5) versus secondary infertility at 15.1% (95% CI: 10.8–21.5). The 36 months overall infertility estimate was 8.8% (95% CI: 6.5–12.3), primary 3.6% (95% CI: 1.2- 9.0), and secondary 9.8% (95% CI: 7.0–14.0).

**Table 3:** Subgroup infertility estimates among women aged 18–44 in Ethiopia based on the current duration approach

	Overall infertility (*N* = 1,577)	Primary infertility (nulliparous *N* = 212)	Secondary infertility (parous *N* = 1,365)
**Infertility at 12 months**	**Prevalence** ^**a**^ **(95%CI)**		
All participants	24.1 (18.8; 34.0)	10.4 (4.1; 26.1)	27.1 (20.2; 39.4)
No education	26.3 (18.7; 48.6)	6.2 (1.4; 53.4)	29.3 (20.6; 54.4)
Primary education or higher	20.4 (13.1; 49.1)	14.1 (5.3; 64.7)	22.8 (13.9; 47.1)
Wants another birth soon	21.0 (13.5; 34.8)	9.7 (3.9; 37.2)	28.8 (9.7; 35.5)
Sexual frequency > 95 times per year	24.3 (18.1; 34.9)	16.8 (6.5; 7.3)	25.5 (17.4; 37.7)
One union	24.4 (18.0; 39.9)	8.8 (1.5; 26.8)	28.3 (20.1; 52.7)
Two or more unions	23.5 (12.1; 66.9)	50.2 (1.7; 72.4)	23.9 (12.6; 64)
**Infertility at 24 months**			
All participants	13.4 (10.1; 18.6)	5.4 (1.9; 13.5)	15.1 (10.8; 21.5)
No education	15.0 (10.3; 23.8)	3.2 (0.7; 39.8)	16.6 (11.2; 27.8)
Primary education or higher	10.6 (6.7; 21)	7 (2.4; 23.3)	12.1 (6.9; 22.3)
Wants another birth soon	12.2 (7.3; 21.7)	4.9 (1.73; 19.3)	18.0 (9.7; 35.5)
Sexual frequency > 95 times per year	13.8 (9.8; 21.2)	9.8 (3.3; 30.1)	14.6 (9.2; 22.6)
One union	13.3 (9.4; 19.8)	4.5 (0.7; 14.8)	15.4 (10.9; 24.3)
Two or more unions	14.1 (6.4; 32.5)	20.6 (0.8; 33)	14.5 (7; 33)
**Infertility at 36 months**			
All participants	8.8 (6.5; 12.3)	3.6 (1.2; 9.0)	9.8 (7.0; 14.0)
No education	10.0 (6.8; 15.7)	2.2 (0.4;33.1)	10.9 (7.3; 16.9)
Primary education or higher	6.7 (4.2; 12.4)	4.4 (1.5; 13.3)	7.7 (4.3; 14.2)
Wants another birth soon	8.3 (4.8; 14.9)	3.1 (1.1; 11.1)	12.5 (6.5; 25.9)
Sexual frequency > 95 times per year	9.2 (6.4; 13.9)	6.8 (2.2; 19.3)	9.6 (5.9; 15.1)
One union	8.6 (6.1; 12.6)	2.9 (0.5; 100)	9.9 (7; 15)
Two or more unions	9.8 (4.3; 22.9)	12.2 (0.5; 20.8)	10 (4.5; 24.5)

^a^ Prevalence based upon a parametric survival function using generalized gamma and 95% CI based on bootstrap methods. The current duration > 36 months were censored at that value

Among women dwelling in rural areas, the 12-month infertility estimate was 26.3% (95% CI 18.7%- 48.6%), determined mainly by parous women 27.0% (95% CI 19.8%- 42.2%). As for the 24-month infertility estimate, parous women with one union had a higher infertility estimate of 15.4% (95% CI 10.9%-24.3%) than nulliparous women 4.5% (95% CI 0.7%- 14.8%). For the 36-month infertility estimate, women with a wealth index of poorest/poorer 9.9% (95% CI 6.4%-16.3%) and middle/richer 8.3% (95% CI 5.2%-15.1%) had comparable infertility estimates with overlapping confidence intervals (CI).

## Discussion

This study is the first in Ethiopia to estimate infertility prevalence from a nationally representative population sample using both the demographic and current duration approaches. The findings demonstrate that both approaches result in significantly different estimations of infertility, with the demographic approach resulting in a lower prevalence of infertility than the CD approach. However, the patterns of primary and secondary infertility were similar, with secondary infertility being higher independent of approach. Our range of estimates is generally in line with studies in other countries, although prevalence estimates of infertility differ due to multiple definitions and study populations.

The study showed that 7.6% of the sample population (an estimated 7.8 million women) were affected by overall infertility using the demographic approach – primary infertility was 1.4%, and secondary infertility was 8.7%. These results corroborate the findings of a multiple-country DHS study, which reported a primary infertility rate of 1.4% and secondary infertility of 9%, respectively, in Ethiopia [35]. The consistent results show the similarities in definition between the two studies and can potentially infer the reliability of the results. Other studies that used the demographic approach in Low and Middle-Income Countries (LMIC) had estimated infertility prevalence from 1.9 to 20.6% for primary and 5–62% for secondary [5, 12, 14, 35]. It is important to note the inconsistencies between the studies, even though they all use a similar approach, which leads to the vast range of prevalence differences of infertility. The EDHS data do not have direct indicators to measure infertility; thus, we used assumptions and proxies to assess the exposure to pregnancy. Additionally, the demographic approach is suitable for detecting trends in a population but unsuitable for clinical purposes as it fails to identify couples needing investigation and treatment. If requested, treatment should commence sooner than five years [21].

Women aged 40–49 had 83% lower primary infertility odds than women aged 20–29, similar to other studies [36, 37]. The lower odds is likely because younger women may not start having children early. On the other hand, older women had a higher chance of secondary infertility. Additionally, the odds of primary infertility increased with age at first cohabitation ≥ 20 years, similar to other studies [36, 38]. Previous studies have shown the widening interval between first cohabitation and first birth in high-income countries and, more recently, in low-income countries [38–40]. Mills et al. argue, “Young adults may also delay childbearing until their income increases, and they can ‘afford’ children, but also to avoid the ‘wage penalty’ of early motherhood.” [41 p. 857] – an argument supported by other researchers as well. Surprisingly, among women with secondary infertility, 60.8% had four or more children. This demonstrates that women with several children have the desire and reproductive right to achieve more children [5], and maybe Ethiopia’s general high fertility rate. To the contrary, a study analyzing infertility trends has shown a decrease in infertility rates in high-income countries. This could be due to the low fertility rate in high income countries, resulting in infertility going unrecognized [42]. For example, if childbearing is complete at a fewer number of children (e.g., 2 kids), infertility that develops later will not be recognized or prevalent at the population level.

Our findings also show that using the demographic approach, women in the richest wealth index had higher odds of primary and secondary infertility than those in the poorest/poorer. In another study, women employed in high-income sectors and earning more had a higher chance of infertility [38]. Wealth index and employment status usually have a linear relation. Women in the richest wealth index might be employed, professional workers prioritizing careers over having children early on; however, this was not shown in our results. Infertility has been associated with greater career progression [5]; however, the reverse may also apply. Additionally, being in more than one union has a higher odds of infertility than one union status in their lifetime, similar to other studies in Ethiopia [31, 37]. One explanation could be that the number of sexual partners increases with the number of unions, which could increase the incidence of sexually transmitted infections leading to infertility. On the other hand, having a child with a partner could increase the chance of being in one union, while having no children could lead to separation, consequently, having more than one union.

Based on the current duration approach, the overall infertility estimate was 24.1% and 13.4% at 12 and 24 months. That is, almost one-fourth of Ethiopian women experienced a delay of more than 12 months to conceive, which is lower than the finding in Nigeria (31.1%) [7]. A study in France that applied the current duration approach estimated infertility to be 24% at 12 months and 11% at 24 months [43]. Our result is comparable with other studies that employed the clinical and epidemiological definition of infertility in different settings [31, 44]. Population-based infertility estimates for shorter durations are limited, making it harder to compare results. The current duration approach can assess TTP using different durations and aligns with the clinical and epidemiological approaches permitting comparison with other studies [7, 43, 45]. Gurunath et al. recommended a clinically relevant definition based on the duration of trying for pregnancy coupled with female age [21]. However, it is also important to note that the clinical – aimed for early detection and treatment – and epidemiologic definitions are inappropriate when making population-based estimates of infertility [12].

Many researchers have suggested the need for a comprehensive infertility definition [12–14, 25], but a consensus is yet to be reached. This crucial methodological consideration further impacts the interpretation of results [29]. The inconsistent estimations of infertility also make developing policies that support preventing and treating infertility challenging [46]. The demographic definition uses assumptions to determine infertility. However, studies vary in the use of pregnancy vs. live birth, the age group to include, the timing of exposure to pregnancy, and the current or lifetime use of contraceptives. Based on our and other similar findings, we recommend that to have comparable results, future researchers and health professionals planning to estimate national infertility rates apply the following proxies based on the nature of the DHS data set: Age group 20–49, five years of pregnancy exposure period, live birth outcome, and lifetime contraceptive use. It is challenging to measure continuous exposure to the risk of pregnancy over the years by using population surveys such as the DHS [14]. However, since the DHS data set is the largest for low-resource regions, questions that directly measure the exposure and intention of pregnancy – variables indicating reproductive capacity – need to be included [7, 47].

This study is not without some limitations. The use of proxies to determine exposure to pregnancy might make estimation unreliable and open for interpretation. Infertility measured based on currently married women may underestimate infertility if couples unable to have children are more likely to dissolve their union than couples with children. Additionally, lack of information from the male partner limits understanding of infertility among men. Although we excluded women who presumably were not exposed to the risk of pregnancy, we could not control for the frequency of sexual intercourse. Fertility desire was assessed only at the time of the survey and did not reflect temporal changes or fluctuations. Our study’s main strength lies in its ability to conduct a direct comparison of two different approaches that use the same data source (DHS) for the same country. This eliminates the need to account for cultural or contextual differences in the comparison of the underlying assumptions of the approaches. However, it should be noted that each approach employs different timelines, eligibility criteria, methods of analysis, and assumptions (Additional file 6). Therefore, it is not feasible to compare each individual component between the two approaches. Furthermore, the two approaches utilized national surveys such as the DHS, which are collected every five years and, therefore, enable monitoring of infertility trends.

## Conclusion

The estimation of infertility using the demographic and current duration approach with EDHS 2016 data resulted in important differences. The findings highlight the importance of definition and methodological congruence for estimating the prevalence of infertility. Regardless of the differences in estimates, infertility is a common condition in Ethiopia and justifies greater attention and resource allocation. Healthcare providers, researchers, and policymakers in Ethiopia need to be aware of the inconsistent and ambiguous definitions and estimation variations when making decisions about the condition.

### Electronic supplementary material

Below is the link to the electronic supplementary material.

Supplementary Material 1

Supplementary Material 2

Supplementary Material 3

Supplementary Material 4

Supplementary Material 5

Supplementary Material 6

## Data Availability

The dataset supporting this study’s findings are available from the DHS program webpage https://dhsprogram.com/ and the codes for the current duration approach driven by Polis and colleagues can be found here (https://osf.io/5jksy).

## Abbreviations

DHS: Demographic and Health Survey
EDHS: Ethiopian Demographic and Health Survey
LMIC: Low- and Middle-Income Countries
TTP: Time To Pregnancy
WHO: World Health Organization
