# Supplementary material for: The varying estimation of infertility in Ethiopia: the need for a comprehensive definition
Source: BMC Womens Health. 2024 May 8;24:280. doi: 10.1186/s12905-024-03118-8 (PMC11077700; doi:10.1186/s12905-024-03118-8)
Supplement: Supplementary file 1 — Supplementary Material 1 [file 12905_2024_3118_MOESM1_ESM.docx]

Women age 20-49

N= 12, 185

Union ≥ 5 years

Not in a union or union< 5 years

N= 4,437

N= 7,748

Not exposed

No birth

≥1 birth

N= 169

N= 7,579

Exposed: Fertile union

Lifetime contraceptive use or no fertility desire

N= 119

N= 50

Not exposed

No contraceptive use and desire a child

Exposed: Infertile union

Additional file 1: Flow diagram of the eligible participants for the Primary infertility sample using the Demographic approach. (N is unweighted )
