# Supplementary material for: The varying estimation of infertility in Ethiopia: the need for a comprehensive definition
Source: BMC Womens Health. 2024 May 8;24:280. doi: 10.1186/s12905-024-03118-8 (PMC11077700; doi:10.1186/s12905-024-03118-8)
Supplement: Supplementary file 2 — Supplementary Material 2 [file 12905_2024_3118_MOESM2_ESM.docx]

Women age 20-49

N= 12,185

Union ≥ 5 years and gave birth to a child ≥5 years ago

No birth or not in a union or union<5years or gave birth to 1^st^ child<5 years ago

N= 5,148

N= 7,037

Not exposed

Last birth ≥5 years ago

Last birth <5 years ago

N= 4,876

N= 2,161

Exposed: Fertile union

Any contraceptive use in the past 5 years or no fertility desire

N= 1,501

Not exposed

No contraceptive use in the past 5 years and desire a child

N= 660

Exposed: Infertile union

Additional file 2: Flow diagram of the eligible participants for the secondary infertility sample using the Demographic approach. (N is unweighted)
