# Supplementary material for: The varying estimation of infertility in Ethiopia: the need for a comprehensive definition
Source: BMC Womens Health. 2024 May 8;24:280. doi: 10.1186/s12905-024-03118-8 (PMC11077700; doi:10.1186/s12905-024-03118-8)
Supplement: Supplementary file 3 — Supplementary Material 3 [file 12905_2024_3118_MOESM3_ESM.docx]

15,683 participants in the EDHS

13,893 did not meet the inclusion criteria

1,790 participants eligible for the CD analysis

213 excluded for potential unrecognized early pregnancy

1,577 participants in the analytic sample

212 nulliparous

1,365 parous

Additional file 3: Flow diagram of the eligible participants for the current duration sample.
