# Supplementary material for: The varying estimation of infertility in Ethiopia: the need for a comprehensive definition
Source: BMC Womens Health. 2024 May 8;24:280. doi: 10.1186/s12905-024-03118-8 (PMC11077700; doi:10.1186/s12905-024-03118-8)
Supplement: Supplementary file 4 — Supplementary Material 4 [file 12905_2024_3118_MOESM4_ESM.docx]

Additional file 4: Characteristics of women in the 2016 EDHS data set by eligibility for the current duration sample

| Characteristic | | Not eligible for CD Group (N=14,106) | Eligible for CD Group (N=1,577) | Total N (N=15,683) | P-value |
| --- | --- | --- | --- | --- | --- |
| **Age at interview (years)** *%* | |  |  |  | <0.001 |
|  | 15-17 | 14.6 | n/a | 13.1 |  |
|  | 18-24 | 27.4 | 15.3 | 26.1 |  |
|  | 25-34 | 33.1 | 40.1 | 33.8 |  |
|  | 35-44 | 17.7 | 44.7 | 20.5 |  |
|  | 45-49 | 7.3 | n/a | 6.5 |  |
| **Marital status** *%* | |  |  |  | <0.001 |
|  | Married | 59.7 | 99 | 63.9 |  |
|  | Living with partner | 1.4 | 1 | 1.3 |  |
|  | Other ^a^ | 39 | 0 | 34.8 |  |
| **Total children ever born** *%* | |  |  |  | <0.001 |
|  | 0 | 34.8 | 12.9 | 32.5 |  |
|  | 1 | 11.9 | 9.9 | 11.7 |  |
|  | 2-4 | 27.7 | 30.2 | 27.9 |  |
|  | 5-7 | 17.6 | 33.5 | 19.3 |  |
|  | 8-16 | 8.1 | 13.6 | 8.7 |  |
| **Residence** *%* | |  |  |  | <0.001 |
|  | urban | 23.2 | 13.5 | 22.2 |  |
|  | rural | 76.8 | 86.5 | 77.8 |  |
| **Women education** *%* | |  |  |  | <0.001 |
|  | no education | 45.5 | 66.9 | 47.8 |  |
|  | primary | 36.2 | 25.4 | 35 |  |
|  | secondary | 12.4 | 4.6 | 11.6 |  |
|  | higher | 5.9 | 3.1 | 5.6 |  |
| **Religion** *%* | |  |  |  | <0.001 |
|  | Orthodox | 44.7 | 31.8 | 43.3 |  |
|  | Other Christian | 24.3 | 23 | 24.2 |  |
|  | Muslim | 29.8 | 42.8 | 31.2 |  |
|  | Other/missing | 1.2 | 2.4 | 1.3 |  |
| **Ever terminated a pregnancy** *%* | |  |  |  | <0.001 |
|  | No | 93 | 84.8 | 92.1 |  |
|  | Yes | 7 | 15.2 | 7.9 |  |
| **Knowledge of modern methods of contraception** *%* | |  |  |  | 0.43 |
|  | Does not know a modern method | 1.7 | 2 | 1.7 |  |
|  | Knows modern method | 98.3 | 98 | 98.3 |  |
| **Contraceptive usage** *%* | |  |  |  | <0.001 |
|  | Never used contraception | 55.4 | 71.2 | 57 |  |
|  | Used contraception | 44.7 | 28.8 | 43 |  |
| **Currently breastfeeding** *%* | |  |  |  | 0.91 |
|  | no | 69.3 | 69.1 | 69.3 |  |
|  | yes | 30.7 | 30.9 | 30.7 |  |
| **Number of partner’s other wives** *%* | |  |  |  | 0.01 |
|  | Non-polygynous | 89.4 | 85.9 | 88.8 |  |
|  | Polygynous union | 10.6 | 14.1 | 11.2 |  |
| **Fertility desire** *%* | |  |  |  | <0.001 |
|  | Wants, Within 2 years | 10.7 | 39.3 | 13.7 |  |
|  | Wants, after 2^+^ years | 38.1 | 17.8 | 35.9 |  |
|  | Wants, Unsure of timing | 10.1 | 3.6 | 9.40 |  |
|  | Undecided | 9.4 | 6.9 | 9.1 |  |
|  | Wants no more | 30.4 | 30.1 | 30.4 |  |
|  | Sterilized | 0.3 | 0 | 0.3 |  |
|  | Declared infecund | 1.1 | 2.3 | 1.2 |  |
| **Husband wants more children than wife** *%* | | 25.2 | 29.7 | 25.9 | 0.02 |
| **Sexual frequency in the last year with most recent partner** *%* | |  |  |  | <0.001 |
|  | 1-20 | 6.6 | 4.2 | 6.2 |  |
|  | 21-40 | 8.2 | 4.4 | 7.5 |  |
|  | 41-60 | 16.2 | 14.9 | 16 |  |
|  | 61-80 | 7 | 8.4 | 7.2 |  |
|  | 80-94 | 1.8 | 1 | 1.6 |  |
|  | 95+ | 60.3 | 67.1 | 61.4 |  |
| **Correct knowledge of the fertile period** *%* | | 23.8 | 22 | 23.6 | 0.24 |

Note: the table presents weighted percentages. ^a^: never in a union, divorced, widowed, and separated

CD: current duration
