# Supplementary material for: The varying estimation of infertility in Ethiopia: the need for a comprehensive definition
Source: BMC Womens Health. 2024 May 8;24:280. doi: 10.1186/s12905-024-03118-8 (PMC11077700; doi:10.1186/s12905-024-03118-8)
Supplement: Supplementary file 5 — Supplementary Material 5 [file 12905_2024_3118_MOESM5_ESM.docx]

Additional file 5: Characteristics of women in the Current Duration sample by parity and fertility intention status

| Characteristic | | Nulliparous  (N=252) | Parous  (N=1,538) | p-value | Does not want another birth soon  (N=990) | Wants another birth soon (N=800) | P-value |
| --- | --- | --- | --- | --- | --- | --- | --- |
| **Age at interview (years)** *%* | |  |  | 0.000 |  |  | <0.001 |
|  | 18-24 | 52.2 | 9.8 |  | 11.3 | 23.4 |  |
|  | 25-34 | 32.2 | 41.2 |  | 39 | 42.2 |  |
|  | 35-44 | 15.7 | 48.9 |  | 49.7 | 34.4 |  |
| **Residence** *%* | |  |  | 0.000 |  |  | <0.001 |
| Urban | | 28.2 | 11.3 |  | 8.7 | 23.4 |  |
| Rural | | 71.8 | 88.7 |  | 91.3 | 76.6 |  |
| **Women education** *%* | |  |  | 0.000 |  |  | <0.001 |
|  | No education | 35.9 | 71.4 |  | 72.1 | 56.2 |  |
|  | Primary | 36.5 | 23.8 |  | 23.4 | 29.6 |  |
|  | Secondary | 14.6 | 3.1 |  | 2.8 | 8.3 |  |
|  | Higher | 13 | 1.7 |  | 1.8 | 5.9 |  |
| **Literacy** *%* | | | | 0.000 |  |  |  |
| Illiterate | | 52.6 | 80.3 |  | 81.3 | 67.4 | <0.001 |
| Literate | | 47.4 | 19.8 |  | 18.7 | 32.7 |  |
| **Religion** *%* | |  |  | 0.39 |  |  | 0.01 |
| Orthodox | | 36.1 | 31.2 |  | 28.2 | 39.3 |  |
| Other Christian | | 26.6 | 22.5 |  | 25.3 | 18.4 |  |
| Muslim | | 36.4 | 43.7 |  | 43.5 | 41.2 |  |
| Other | | 1 | 2.6 |  | 3 | 1.1 |  |
| **Ever terminated a pregnancy** % | |  |  | 0.29 |  |  | 0.3 |
| Yes | | 11.9 | 15.7 |  | 14.2 | 17.2 |  |
| No | | 88.1 | 84.3 |  | 85.8 | 82.8 |  |
| **Currently breastfeeding** *%* | |  |  | 0.000 |  |  | <0.001 |
| No | | 1 | 64.6 |  | 58.9 | 90 |  |
| Yes | | 0 | 35.4 |  | 41.2 | 10 |  |
| **Number of partner’s other wives** *%* | |  |  | 0.17 |  |  | 0.01 |
| Non-polygynous | | 90.8 | 85.2 |  | 83.8 | 90.2 |  |
| polygynous | | 9.2 | 14.8 |  | 16.2 | 9.8 |  |
| **Fertility desire** *%* | |  |  | 0.000 |  |  | <0.001 |
| Wants, within 2 years | | 78.8 | 33.5 |  | 9.5 | 100 |  |
| Wants, after 2^+^ years | | 5.3 | 19.6 |  | 26.5 | 0 |  |
| Wants, unsure timing | | 5 | 3.4 |  | 5.3 | 0 |  |
| Wants, undecided | | 1.5 | 7.7 |  | 10.2 | 0 |  |
| Wants no more | | 4 | 34 |  | 45 | 0 |  |
| Declared infecund | | 5.4 | 1.9 |  | 3.5 | 0 |  |
| **Husband wants more children than wife** *%* | | 26.7 | 30.1 | 0.53 | 31.5 | 26 | 0.17 |
| **Sexual frequency in last year with most recent partner** *%* | |  |  | 0.19 |  |  | 0.5 |
| 1-20 | | 8.1 | 3.6 |  | 4.0 | 4.6 |  |
| 21-40 | | 6.4 | 4.1 |  | 4.3 | 4.6 |  |
| 41-60 | | 18.5 | 14.4 |  | 13.7 | 17.4 |  |
| 61-80 | | 6.9 | 8.6 |  | 8.4 | 8.4 |  |
| 81-94 | | 0.8 | 1.0 |  | 1.3 | 0.3 |  |
| 95+ | | 59.3 | 68.3 |  | 68.3 | 64.7 |  |
| **knowledge of the fertile period** *%* | |  |  | 0.44 |  |  | 0.7 |
| Correct | | 25 | 21.5 |  | 21.5 | 23 |  |
| Incorrect | | 75 | 78.5 |  | 78.5 | 77 |  |

Note: the table presents weighted percentages.
