# Supplementary material for: The varying estimation of infertility in Ethiopia: the need for a comprehensive definition
Source: BMC Womens Health. 2024 May 8;24:280. doi: 10.1186/s12905-024-03118-8 (PMC11077700; doi:10.1186/s12905-024-03118-8)
Supplement: Supplementary file 6 — Supplementary Material 6 [file 12905_2024_3118_MOESM6_ESM.docx]

| Additional file 6: Summary of the Demographic and current duration approaches to measuring infertility using data from the Ethiopian Demographic Health Survey. | | |
| --- | --- | --- |
|  | Demographic approach | Current duration approach |
| Description | Infertility is estimated by the absence of live births among women living with a partner/married and who are exposed to pregnancy.  Exposure is inferred by the proxies of living with a partner/married, having a fertility desire, and not using contraceptives from the EDHS survey. | Infertility is defined as the absence of pregnancy after a certain period of time trying to conceive, known as time to pregnancy (TTP).  Includes women ‘at risk’ of pregnancy at the time of the interview and certain assumptions.  The time is calculated from the start of the pregnancy attempt/live birth to the interview date. |
| Questions extracted from the data set. | The questions extracted are focused on specific demographics, including age (20-49), relationship status (only those in a union or married), contraceptive use (those who have no contraceptive history before for primary infertility and those who have not used it for the past five years for secondary infertility), those who have a desire to conceive, and history of live birth (those who have never given birth before for primary infertility and those who have given birth in the past but not in the last five years for secondary infertility). | For those at risk of pregnancy, questions are based on age (18–44), married or cohabitating, sexually active within the past four weeks, not currently using contraception, date of last live birth or pregnancy, duration of postpartum abstinence and amenorrhea for the most recent live birth, and date of interview. |
| Objectives of the study | Generate population-based infertility estimates.  Determine factors associated with infertility. | Estimates fecundity (i.e., the biological capacity for reproduction) as a measure of infertility prevalence.  Subgroup analyses to understand infertility rates and risk factors in different groups. |
| Duration cut-off to determine infertility | 60 months | 12 months  24 months  36 months |
| Methods of analysis | Binary logistic regression | Parametric survival analysis |
